# Sex Differences in the Genetic Risk for Alcoholism

**Published:** 2002

**Authors:** Carol A. Prescott

**Affiliations:** Carol A. Prescott, Ph.D., is an associate professor of psychiatry at the Virginia Institute for Psychiatric and Behavioral Genetics and co-director of the Virginia Adult Twin Study of Psychiatric and Substance Use Disorders, both at the Medical College of Virginia of Virginia Commonwealth University, Richmond, Virginia

**Keywords:** gender differences, genetic linkage, hereditary factors, risk factors, twin study, adoption study, epidemiological indicators, etiology, molecular genetics, AOD (alcohol and other drug) use susceptibility, AOD dependence potential, comorbidity, alcoholic beverage

## Abstract

One of the characteristics influencing a person’s risk for alcoholism is his or her sex, and various factors may contribute to sex differences in risk. Adoption studies have provided some evidence of possible sex differences in the heritability of alcoholism, but overall the findings have been inconclusive. Twin studies have consistently supported the role of genetic risk factors in the heritability of alcoholism in men, and shared environmental factors also play a role in the familiality of alcoholism among women. In addition, sex differences exist in the patterns of transmission of alcoholism between family members. However, the genetic epidemiology research conducted to date on this issue has several limitations, some of which may be resolved by future molecular genetic studies.

One of the factors associated with a person’s risk for alcoholism^1^ is his or her sex. In this article, the term “sex” is used in both its biological sense (i.e., as a variable based on genetic differences between males and females) and its cultural meaning (i.e., in the sense of gender roles). Sex is associated with both biological risk factors (e.g., sex-specific hormone systems) and cultural risk factors (e.g., social expectations about how men and women use alcohol). An understanding of the mechanisms influencing sex differences in risk can help illuminate not only the differences in men’s and women’s drinking behavior and related problems but also the biological and cultural bases for variability within each sex.

Sex differences in the factors underlying the development of alcoholism (i.e., its etiology) may be manifested as differences in prevalence, in the magnitude of genetic influences, and in the sources of genetic influences (i.e., sex-specific transmission of genetic risk factors). Studies in many cultures have found that the prevalence of alcoholism and heavy drinking generally is higher among men than among women. Both cultural and biological explanations have been invoked to account for this difference, but the mechanisms remain unclear. Moreover, differences in prevalence may arise even if the mechanisms underlying alcoholism development do not differ between the sexes. In this case, the same genetic factors could predispose men and women to alcoholism, but other sex-specific genetic and/or environmental factors could influence whether alcoholism develops in a given person.

The magnitude of genetic influences on alcoholism risk may also be sex specific. Evidence from twin and adoption studies of alcoholism in males has consistently supported the existence of moderate genetic influences, accounting for about half of the population variation in liability to develop alcoholism (Prescott 2001). However, as will be described in this article, the evidence regarding the role of genetic factors in alcoholism in women has varied across studies. Such sex differences in the magnitude of genetic influence could arise from the interactions between genes associated with alcoholism risk and other physiological processes. For example, numerous physiological differences between men and women in the rate of alcohol absorption and metabolism are likely to be genetic in origin and may influence the development of alcoholism.

Another potential manifestation of sex differences in alcoholism risk is the presence of sex-specific etiological factors. In this case, the risk that a relative of an alcoholic will develop alcoholism is greater when the relative and the alcoholic are of the same sex than when they are of different sexes. Sex-specific differences in etiology could be caused by genes that exhibit different levels of activity in men and women or which are modified by other sex-specific genetic or environmental factors to make male and female relatives less similar. These sex-specific etiological differences also could arise from cultural processes—for example, whether girls are more likely to model their drinking behavior after their mothers and boys after their fathers.

This article explores the various mechanisms that may contribute to sex differences in the genetic risk for alcoholism. After reviewing genetic epidemiology studies of alcoholism that assess genetic versus environmental contributions to alcoholism risk, the article evaluates various explanations for apparent sex differences in heritability, summarizes evidence for sex differences in patterns of transmission, and discusses the limitations of such research. The article then briefly addresses molecular genetic studies before examining potential applications of research on sex differences.

## Genetic Epidemiology Studies of Alcoholism

More than 50 family studies have shown that alcoholism runs in families. Those studies found that first-degree relatives (e.g., parents, children, or siblings) of treated alcoholics had a two to four times higher risk of being alcoholics themselves than did relatives of nonalcoholics (Cotton 1979). Studies of intact families cannot determine, however, whether this familiality arises because family members share genes or because they share environments. This question must be resolved using genetic epidemiology studies—studies using genetically informative samples, such as twins or adoptees and their biological and adoptive relatives.

The goal of genetic epidemiology research is to understand sources of variability in the risk of developing a disorder such as alcoholism. It is usually assumed that alcoholism is a multifactorial disorder in which a combination of genetic and environmental factors contributes to the risk of developing the disorder. People differ in their level of risk for the disorder, and individual risk probably arises from three sources:

- *Additive genetic variation* (also called heritability), which is based on numerous genes that exist in multiple variants (i.e., alleles), each of which may have a different effect. A person’s specific combination of alleles of those genes determines his or her genetic risk. The role of such genetic influences is usually not measured directly but is inferred by comparing degree of similarity in a characteristic or behavior among different classes of relatives.
- *Shared environment*, which includes all environmental factors that make members of the same family similar to one another (and different from members of other families), including family environment; social class; schools; and, for twins, the intrauterine environment.
- *Specific environment*, which includes all remaining environmental factors not shared by family members.

The following sections discuss the findings of adoption and twin studies with respect to sex differences. For more detailed descriptions of individual studies reviewed here, the reader is referred to reviews by McGue (1994) and Prescott (2001).

### Adoption Studies

Adoption studies allow researchers to separate the effects of genetic and environmental factors because adoptees receive their genetic heritage from one set of parents and their rearing environment from another set. Given some assumptions (e.g., absence of intrauterine effects on risk for developing alcoholism and no correlation between the adoptive and biological family environments for factors that influence alcoholism risk), the degree to which adoptees resemble their biological relatives is a direct measure of genetic influence. Conversely, the degree to which adoptees resemble their adoptive relatives is a measure of the influence of family environment.

Five adoption studies of alcoholism in males and four in females have included adequate sample sizes to evaluate genetic influences on alcoholism. Three of the studies were conducted in Europe and used government registries to identify adoptees whose biological parents had records of alcoholism treatment or alcohol-related offenses. Two other studies were conducted in Iowa among adoptees for whom alcoholism status was determined in adulthood using psychiatric evaluations as well as public records. In these two studies, the adoptees were classified as “family history negative” if neither biological parent showed evidence of alcoholism and as “family history positive” if at least one biological parent showed evidence of alcoholism.

In all five studies of male adoptees, participants with a positive family history were at significantly higher (i.e., 1.6 to 3.6 times greater) risk for alcoholism than were participants with a negative family history, suggesting a substantial genetic contribution to the risk for alcoholism. The studies of female adoptees, in contrast, obtained mixed results, with only two of the four studies finding a significantly increased risk of alcoholism among females with a positive family history compared with females with a negative family history. These results provided some evidence of possible sex differences in heritability, but are inconclusive because of the small numbers of alcoholic female adoptees in the studies.

### Twin Studies

Studies comparing the resemblance of identical and fraternal twins also allow researchers to estimate genetic and environmental contributions to the risk for alcoholism. Identical twins share all of their genetic factors and are equally exposed to the shared environmental factors. Conversely, fraternal twins (like ordinary siblings) share on average only one-half of their genetic variation but are also equally exposed to the shared environmental factors. Consequently, if identical twins are more similar to each other in their risk for alcoholism than are fraternal twins, one can assume that genetic factors contribute to the vulnerability to alcoholism, with the extent of the resemblance of identical twins indicating the extent of the contribution of genetic factors. However, if identical twins are no more similar to each other in their risk for alcoholism than are fraternal twins, this indicates that alcoholism runs in families because of environmental factors shared by family members. The extent of dissimilarity of identical twins indicates the extent of the influence exerted by specific environmental factors and personal experiences on the risk for alcoholism.

The results of 11 major twin studies of alcoholism have varied with the methods used to identify participants. Four studies identified affected people (i.e., probands) through treatment settings or archival records and then assessed the clinical status of their co-twins through individual followup. Three of these four studies obtained evidence for significant genetic influences on alcoholism in men. In the three treatment-based studies that included women, the evidence for genetic influences was weaker, and some evidence suggested that environmental factors also contributed to the familial similarity for alcoholism. However, because of relatively small numbers of female twin pairs participating, these studies had limited ability to detect sex differences.

Four other studies matched twin registries against archival records (e.g., government records of alcohol offenses, military records, and medical records) to categorize twin pairs as to whether both twins, only one twin, or neither twin had records suggesting alcoholism. In these studies, the prevalence of alcoholism generally was low, suggesting that the samples were selected for severe cases. This means that alcoholics who were not hospitalized would have gone undetected, leading to underestimates of how many twin pairs were similar for alcoholism. Two of the four studies included female twins; however, in these samples the prevalence of alcoholism was less than 1 percent overall. As a result, these studies have a very limited ability to distinguish the extent to which genetic and environmental factors caused familial resemblance and whether sex differences existed.

Three studies used population-based twin registries and recruited participants regardless of whether they were alcoholic or not. All participants were then assessed for history of alcoholism through personal interviews. One of these studies, the Virginia Adult Twin Study of Psychiatric and Substance Use Disorders (VATSPSUD), includes male, female, and male–female twin pairs from Virginia and is described in more detail in the following paragraphs. A second population-based twin study, which also included male, female, and opposite-sex pairs, is based on a volunteer registry in Australia. The third study included only male twin pairs who served in the U.S. military during the Vietnam War era.

All three of these studies found that, among male twin pairs, identical twin pairs were more similar for alcoholism than fraternal pairs, indicating a significant genetic contribution to risk for alcoholism in males. The two studies that included female twin pairs yielded heritability estimates for women similar to those for men. The one difference between these studies was for the results based on the opposite-sex pairs. Whereas the VATSPSUD study concluded that males and females have only partially overlapping genetic risk factors for alcoholism, the study conducted in Australia found evidence that the risk factors in males and females are the same.

#### The Virginia Adult Twin Study of Psychiatric and Substance Use Disorders

The VATSPSUD is a longitudinal study of adult twin pairs conducted at Virginia Commonwealth University. It was initiated in 1988 as a study of female twin pairs by Kenneth Kendler and colleagues (Kendler et al. 1992) and has been funded by the National Institute of Mental Health, the National Institute on Alcohol Abuse and Alcoholism, and the National Institute on Drug Abuse. To date, the original study of female twins has completed four rounds of interviews with the twin pairs as well as interviews with their parents. A parallel study of male and opposite-sex twin pairs, begun in 1993, to date has completed two waves of interviews with both groups, and a third wave of interviews with male twin pairs is scheduled to be completed in 2003. All three groups of twin pairs are white twins born between 1934 and 1975 who were drawn from a registry of multiple births in the Commonwealth of Virginia. Overall, the study comprises more than 8,700 people, including more than 3,750 complete twin pairs. Comparisons with national and regional census data indicate that the twin samples are broadly representative of the white population of native-born Virginians in this age range.

Kendler and colleagues (1992) published the results on twin resemblance for alcoholism based on the first wave of interviews with the female twin pairs in the VATSPSUD. Heritability estimates ranged from 0.50 to 0.61 for several definitions of alcoholism. This means that 50 to 60 percent of the variation in risk for alcoholism results from genetic factors. In 1999, Prescott and colleagues published an extension of this work that included the new samples of male and opposite-sex pairs and an extensive re-evaluation of the female sample. Heritability estimates ranged from 0.51 to 0.66 for all definitions of alcoholism studied and were of similar magnitude for males and females. Little evidence suggested that common environmental factors contribute to twin pair similarity for alcoholism.

#### Summary of Twin Study Findings

In summary, twin studies of males have consistently found significantly more resemblance in identical twins with respect to alcoholism than in fraternal twins, implying an important role for genetic factors in the vulnerability to alcoholism. The heritability estimates in most studies suggest that genetic variability accounts for 50 to 60 percent of the variation in risk for alcoholism in males. Studies of female twins, in contrast, are less consistent. Studies based on samples identified through treatment settings suggest that familiality of alcoholism among women can be attributed to shared environments rather than (or in addition) to genetic factors. Studies using population-based twin registries have found the genetic influences on alcoholism to be of similar magnitude in males and females.

## Explanations for Apparent Sex Differences in Heritability

The results from twin and adoption studies fairly consistently support a moderate to strong familiality of alcoholism in both men and women. For men, most of this familiality can be attributed to genetic factors, whereas the proposed sources of the familiality among women vary across studies. One possible explanation for this apparent sex difference in the sources of familiality is that the studies have limited ability to distinguish between genetic and shared environmental effects because of their small sample sizes. Even within studies, different heritability estimates can be obtained depending on the diagnostic definition used (e.g., alcohol dependence versus alcohol abuse). Consistent with this explanation, Heath and colleagues’ reanalyses (1998) of these studies suggest that some of the reported sex differences are not statistically significant. Other possible explanations for the variability in findings are discussed in the following sections.

### Etiological Heterogeneity

Another possible explanation for sex differences in genetic influences on alcoholism is etiological heterogeneity—differences among subgroups (e.g., men and women) in the genetic factors that influence the development of alcoholism. Most current theories assume that multiple genes contribute to the risk for alcoholism, and different people have different sets of predisposing genes. However, the existence of heterogeneity is difficult to detect using genetic epidemiology studies because the predisposing genes are not yet known. Therefore, researchers cannot study those genes directly but must infer the presence of heterogeneity when heritability differs in subgroups.

For example, two twin studies of males found evidence for greater heritability among males with early-onset alcoholism than among males with late-onset alcoholism (McGue et al. 1992; Caldwell and Gottesman 1991). The researchers theorized that, of the two types, late-onset alcoholism was clinically and etiologically more similar to alcoholism in women. However, preliminary analyses from the Virginia and Australian twin samples did not support this distinction. Furthermore, even if early-onset alcoholism was associated with greater heritability and, consequently, with an increased risk of alcoholism in relatives compared with late-onset alcoholism, this would not necessarily indicate distinct etiologies for the two types of alcoholism. Rather, early onset of the disorder could reflect greater liability (e.g., a higher number of risk factors) in the affected people, which also would be expected to be associated with an increased risk in relatives.

Other attempts to find etiologically distinct subtypes of alcoholism have used patterns of clinical symptoms (e.g., Babor et al. 1992). However, typologies tested in genetically informative samples suggested that such “types” are distinguished primarily by disorder severity rather than by distinct patterns of clinical features. In summary, although it seems likely that there are different etiologies that lead to the clinical manifestations of alcoholism, researchers do not yet have strong evidence that these etiologies are associated with genetic differences between subtypes. Identification of genetically distinct subtypes, and an understanding of the role of sex in their etiology, may require the identification of specific genes that predispose to alcoholism.

### Ascertainment Method

A third possible explanation for apparent sex differences lies in the fact that the means by which cases were identified (i.e., case ascertainment) varied from study to study. As noted above, the four twin studies that reported lower heritability of alcoholism in women than in men all used treatment-ascertained samples. Studies using such treatment samples or archival data of alcoholism-related records are likely to be nonrepresentative in several ways. For example, such studies may include cases that are characterized by greater severity or comorbidity (that is, co-occurrence with other disorders) than alcoholism in the general population because patients with these characteristics may be most likely to seek treatment or be included in other alcoholism-related records. This selectivity may be particularly severe in women, because treatment-based twin studies have reported that more than 60 percent of alcoholic women had a comorbid disorder, a much higher proportion than among men in the same samples. Common comorbid disorders include depression, other drug abuse and dependence, and antisocial personality disorder.

If a person’s decision to enter treatment is not simply dependent on his or her genetic vulnerability to alcoholism, drawing study participants from among alcoholics in treatment could result in an unrepresentative sample and yield incorrect estimates of genetic influences. Results from two U.S. twin studies suggest that treatment seeking is not simply determined by a person’s liability to have alcoholism (True et al. 1996). For example, having comorbid depression or having family members who have received treatment could influence a person’s decision to seek treatment. Consequently, the use of a treatment-ascertainment design may result in increased evidence for shared environmental effects among both male and female twins and could be one explanation for why studies of female twins using this design obtain lower heritability estimates than studies using population-based samples (Prescott and Kendler 2000*b*).

## Evidence for Sex Differences in Patterns of Transmission

Several of the key questions about sex differences in the etiology of alcoholism can be addressed only by comparing the resemblance in male–female relative pairs with that in same-sex relative pairs. Four twin studies, two adoption studies, and seven family studies have performed systematic diagnostic assessments and have included information on resemblance for all types of pairs (i.e., male–male, female–female, and male–female). The results of these studies can be used to analyze mechanisms underlying sex differences.

Prescott (2001) conducted a meta-analysis of the data from these studies to test two alternative explanations for the development of sex differences. The first explanation posits the existence of sex-specific thresholds—that is, that women on average need to cross a higher threshold (i.e., have a higher level) of inherited risk than men to develop alcoholism. This hypothesis would account for the lower frequency of alcoholism in women than in men. Moreover, the hypothesis predicts that relatives of female alcoholics are at higher risk of developing alcoholism than are the relatives of male alcoholics. Data from family, adoption, and twin studies based on treatment samples all show evidence of a higher risk associated with having an alcoholic female relative than with having an alcoholic male relative. Twin studies based on population samples showed less increase in risk, possibly because of differences in either severity or etiology between alcoholic women who had been in treatment and alcoholic women in the general population. Across all studies, relatives of female alcoholics had an approximately 40 percent greater risk of alcoholism than relatives of male alcoholics.

The second possible explanation for sex differences is the existence of sex-specific transmission of risk factors— that is, that family members are at greater risk of developing alcoholism if they are the same sex as the alcoholic relative. Researchers conducting the previously mentioned VATSPSUD project found evidence consistent with such a sex-specific transmission. Their data suggested that the transmission of risk factors across sexes was only half as strong as within-sex transmission (Prescott et al. 1999). The evidence regarding sex-specific transmission in other studies is inconclusive, however, because most studies include too few alcoholic women to evaluate this hypothesis thoroughly.

Overall, the evidence from twin and adoption studies suggests that the differences in transmission patterns result from genetic rather than cultural transmission effects between family members. Such genetic transmission effects could be caused by differences in the activity of genes that are located on the sex chromosomes (i.e., the X and Y chromosomes) or in the activities of other genes that are triggered by biological differences between the sexes (e.g., hormone levels).

## Limitations of Research on Sex Differences in Alcoholism

The ability to detect sex differences in the etiology of alcoholism is limited by several methodological problems. Probably the most serious problem arises from the small numbers of affected women in most studies. It is difficult to overcome this limitation because twin and adoption studies have already attempted to identify all affected people in entire countries or regions. Even the VATSPSUD project, which was designed specifically to test for sex differences and includes more than 1,400 pairs of opposite-sex twins, still has limited power for some questions. It is unlikely that future studies using twin or adoption designs will have greater sample sizes. To address this limitation, researchers may have to await the identification of the specific genes and physiological mechanisms involved in the development of alcoholism. These processes could then be studied to determine if they differ between men and women and if this explains the differences observed clinically.

Analyses of sex differences are also limited by the fact that estimates of genetic and environmental effects assume that all forms of alcoholism have the same etiology. However, different genetic factors may lead to the development of different subtypes of alcoholism. As described previously, some researchers have addressed this issue by investigating whether heritability differs for alcoholism subtypes that are characterized by a specific age of onset or certain clinical features. The results of these analyses, however, have been equivocal.

As mentioned before, other limitations are associated with the method of ascertaining cases. Thus, studies using subjects undergoing alcoholism treatment may include only a small fraction of alcoholics residing in the community. This selectivity can be a problem particularly if sex differences exist in the detection of alcoholism (i.e., in how researchers and treatment providers identify female and male alcoholics) or in treatment entry of alcoholics. Studies using clinical interviews in community samples identify a larger proportion of alcoholics than do treatment studies. However, one can argue that the definition of alcoholism in these interviews is too broad because it includes many people who experience a single transient episode of alcohol-related problems and recover on their own.

Another limitation is that studies often include relatives who are in their twenties or thirties and still at risk for developing alcoholism. Therefore, the results regarding similarities among relatives might change if currently unaffected people were assessed at a later time. Studies examining different degrees in similarity in different age groups typically have not found that older relative pairs are more similar, as would be expected if under-diagnosis in younger age groups were a problem. However, the statistical power of these tests is limited.

Finally, most of the information available concerns only the etiology of alcoholism in white populations. Only one sample (Caldwell and Gottesman 1991) has included more than 10 percent nonwhites. The heritability estimates from this study differed by ethnicity but the differences did not reach statistical significance because of the small sample sizes of different ethnic groups.

Despite all these limitations, however, it is clear that sex is an important factor to consider in the study of the etiology of alcoholism.

## Molecular Genetic Studies

The goal of molecular genetic studies is to identify specific genes involved in the development of alcoholism. Researchers have already conducted several large-scale genetic family studies (e.g., Reich et al. 1998), and other studies are under way to search the entire human genetic material (i.e., the genome) for genes that increase the risk for alcoholism. Many other studies have analyzed candidate genes— regions known to influence alcohol metabolism (e.g., genes encoding the enzymes that break down alcohol) or known to be involved in brain activity (e.g., genes coding for various brain chemicals [i.e., neurotransmitters])— for their possible association with alcoholism in clinical populations. Thus far, few reports suggest significant sex differences in the roles of specific genes. However, most studies have included relatively few alcoholic women, limiting the ability to test for sex effects.

Some evidence from molecular genetic studies suggests that there may be etiologically distinct subtypes of alcoholism. For example, people who have alcoholism characterized by aggressivity or antisocial personality are more likely to carry a gene that is associated with increased activity of the neurotransmitter serotonin in the brain (e.g., Preuss et al. 2001). Because this alcoholism subtype is found mostly in men and only rarely in women, this could provide one explanation for why the sexes differ in genetic risk factors for alcoholism.

## Applications of Research on Sex Differences

One explanation for the existence of sex differences in risk for alcoholism is that males and females differ on the degree to which they experience the various risk factors for alcoholism. This means that sex is not a risk factor in itself but is merely correlated with some of the processes underlying alcoholism. For example, the sexes may differ in the mechanisms underlying the co-occurrence of alcoholism with other disorders, and they may differ in factors that moderate or mediate genetic influences on alcoholism. These issues are being addressed by several recent twin and adoption studies, as discussed in the following sections.

### Genetic Influences on the Occurrence of Comorbid Disorders

Alcohol use disorders are frequently accompanied by other emotional disorders (e.g., depression and anxiety) and other drug use disorders. Comorbid disorders have been estimated to occur in as many as 80 percent of clinical populations and 50 percent of community samples (Kessler et al. 1997). The etiology of this comorbidity is of theoretical and clinical importance because these coexisting disorders complicate treatment and may alter the client’s prognosis. Male and female alcoholics differ in their patterns of comorbidity, with women having higher rates of comorbid anxiety and affective disorders and males having higher rates of comorbid abuse of other drugs, conduct disorder, and antisocial personality disorder (Kessler et al. 1997). Because of these differences, a better understanding of the causes of comorbidity may help researchers uncover the basis for sex differences in the genetic risk for alcoholism.

**Figure 1:**
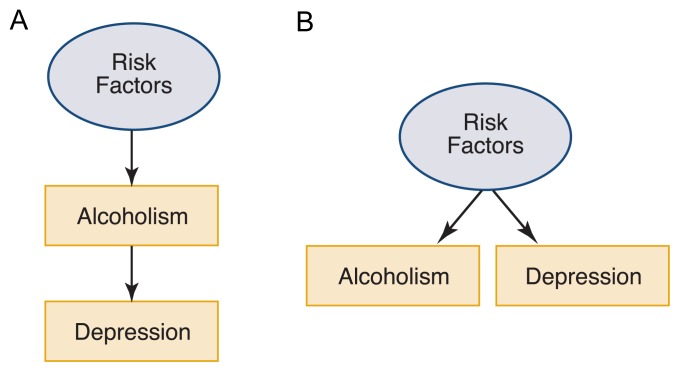
Schematic models of possible mechanisms underlying comorbidity between alcoholism and other psychiatric disorders. Circles indicate unobserved risk. Boxes indicate observed clinical outcomes. (A) The model of phenotypic association posits that the presence of one disorder increases the risk of acquiring the second disorder. For example, a person with alcoholism could be at increased risk of developing depression. (The reverse scenario—that a person with depression is at increased risk of alcoholism—is also possible but is not shown in this figure.) (B) According to the model of correlated causes, the same risk factors contribute to development of both disorders.

There are several explanations for why two disorders may co-occur. One explanation—known as phenotypic association—suggests that the presence of one disorder (e.g., alcoholism) increases the risk for another disorder (e.g., depression) because of physiological processes or as a reaction to the psychosocial problems that accompany alcoholism (see figure 1A). (The reverse is also possible—that is, alcoholism can result from depression because a person may use alcohol in an attempt to deal with the depression.) A second explanation for comorbidity proposes the presence of correlated causes (see figure 1B). According to this model, alcoholism and depression may occur together because the risk factors for both disorders are the same (i.e., are correlated).

Depression provides an example of how genetic factors may be associated with sex differences in risk for a disorder. During adolescence through mid-adulthood, females have much higher rates of depression than males, whereas these differences are much less pronounced during other parts of the life span. This observation, combined with results from experimental research with animal models of depression, suggests that the level of circulating estrogen (which is genetically influenced) plays a role in the higher incidence of depression in females (Halbreich and Kahn 2001).

**Figure 2:**
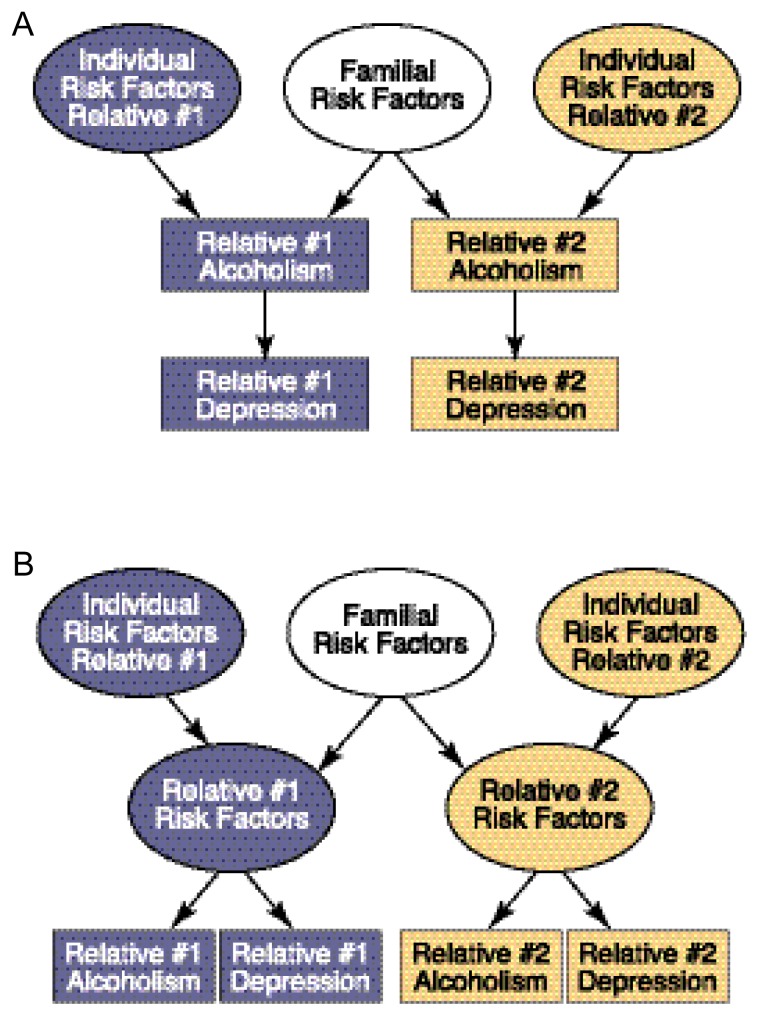
Schematic models of mechanisms underlying comorbidity between alcoholism and other psychiatric disorders among relatives. (A) According to the phenotypic association model, alcoholism develops because of a combination of individual and familial risk factors; moreover, alcoholism increases the risk of depression. In this scenario, relatives of an alcoholic (relative #1) are at increased risk for depression only if the relatives also have alcoholism. (B) Under the correlated causes model, individual and familial risk factors contribute to a general risk of developing alcoholism or depression. In this case, relatives of an alcoholic would be at increased risk for either alcoholism or depression.

The causes of comorbidity between alcoholism and depression have been investigated extensively using family studies. The usual strategy in these studies is to identify probands affected by one or both disorders and examine the prevalences of depression and alcoholism alone or in combination among the proband’s biological relatives. These prevalences are then compared for different types of probands (e.g., depressed, alcoholic, and depressed plus alcoholic probands as well as unaffected control subjects) to determine their fit with possible models of comorbidity. For example, if alcoholism increases the risk of depression as stated by the phenotypic association model, only risk factors for alcoholism would be transmitted across family members, and these risk factors would only indirectly influence the risk for depression (see figure 2A). In this case, if a proband had both alcoholism and depression, a relative of the proband would be expected to be at increased risk for depression (compared with the relatives of people without alcoholism or depression) only if he or she also had alcoholism. Conversely, according to the correlated causes model, the familial risk would be nonspecific and could increase the risk for either alcoholism or depression (see figure 2B). In this case, if a proband had both alcoholism and depression, a relative would be at increased risk of either depression or alcoholism.

Although numerous studies of this issue have been conducted, it is difficult to generalize about their results because of variation in the types of probands studied, in the method of data collection (e.g., by personal interview or from a family informant), and in other methodological features (for a review, see Swendsen and Merikangas 2000). In general, studies using alcoholic probands found that relatives were not at increased risk for depression unless the proband also had depression. Moreover, studies of probands treated for alcoholism with or without comorbid depression found that the relatives of probands with both alcoholism and depression were not at increased risk for alcoholism compared with relatives of probands who had alcoholism alone. These findings are consistent with a phenotypic association between the two disorders (see figure 2A) where the presence of alcoholism increases the risk for depression.

Family studies based on probands with primary depression (i.e., people in whom alcoholism is not present or is considered the result of depression) have yielded mixed findings. Some studies found no increase in alcoholism among relatives unless the probands were also alcoholic. This finding is consistent with the phenotypic association—that is, depression increased risk for alcoholism only in the depressed person but not among nondepressed family members, similar to the model in figure 2A but with depression leading to alcoholism rather than vice versa. Other studies found increased rates of alcoholism among relatives of depressed probands; in some cases, however, this was true only for relatives of female probands.

The family study literature has several limitations. One limitation is that probands recruited in treatment settings are likely to have more severe alcoholism and greater comorbidity and may have different underlying causes than probands recruited from the general population. In fact, studies based on U.S. population samples suggest that depression and alcoholism have correlated causes (Prescott and Kendler 2000*a*).

Another limitation is that family studies cannot distinguish whether vulnerability is transmitted through genetic or environmental pathways. This issue can be addressed using twin and adoption studies; however, the results of such studies have been equivocal. Of four adoption studies, two found weak evidence of increased depression among adopted-away children of alcoholic parents, one found no increased depression, and another found increased alcoholism among biological relatives of depressed adoptees. Goodwin and colleagues (1977) reported increased depression in the daughters of alcoholics only when they were raised by alcoholic adoptive parents, suggesting a possible interaction between genetic and environmental factors. Results from three twin studies based on clinical samples suggest that depression and alcoholism are phenotypically associated. However, a recent molecular genetic study identified a region on chromosome 1 that was associated with an increased risk for both alcoholism and depression, consistent with the correlated causes model in which the same gene(s) contribute to both disorders (Nurnberger et al. 2001).

Researchers in the VATSPSUD project also addressed these issues by studying the causes of comorbidity of major depression and alcoholism (Prescott and Kendler 2000*a*). Their analyses found that people with major depression were at significantly increased risk for alcoholism.^2^ Moreover, a history of major depression in one twin increased the co-twin’s risk for alcoholism to a significant degree among identical twins, to a lesser degree among same-sex fraternal twins, and not at all among opposite-sex twins. The results of the statistical analyses were most consistent with the correlated causes model, which posits that comorbidity occurs because the genetic and specific environmental sources of liability for depression overlap with those for alcoholism. This overlap was statistically significant only within a sex but not across sexes, indicating that the comorbidity appears to result from sex-specific genetic and environmental risk factors. Unlike previous hypotheses, these results suggest that the factors underlying depression in women appear to differ from the factors underlying alcoholism in men.

It is important to note that these findings do not point to one definitive mechanism underlying comorbidity; in fact, they are what would be expected if all three processes were operating in different subsets of the population. It seems reasonable that for some people, depression is a consequence of alcoholism; for others, alcoholism is caused by depression; and for still others, both disorders result from a shared, familially transmitted vulnerability to both disorders.

### Factors That Modify Genetic Liability

Another promising application of genetic epidemiology studies, including studies of sex differences, is the identification of factors that may influence the expression of genetic liability for alcoholism. Researchers are studying two types of influences: modifiers and mediators (discussed in the following section). Modifiers are genetic or environmental factors that interact with genetic risk factors to increase or decrease the risk for alcoholism. Research into these modifying factors has important implications for the prevention of alcoholism. For example, if people can be identified as being at risk for alcoholism based on their genetic backgrounds (e.g., because they carry genes known to increase risk or because they have alcoholic family members), knowledge of modifying factors would permit early interventions.

Investigators have conducted extensive analyses among adoptees and their families in Iowa to identify environmental factors that may increase the risk for alcoholism, other drug abuse, and antisocial personality disorder. These analyses found that having an adoptive family member with alcohol problems increased the risk for alcohol abuse among adopted children whose biological parents were alcoholics (Cadoret et al. 1985). This was particularly true for adoptees raised in rural communities, suggesting shared environmental factors may be less influential in more diverse, urban settings. Additional analyses suggested that environmental factors act differently in males and females to influence the risk for alcoholism and related disorders (e.g., Cadoret et al. 1999). For example, in the presence of environmental stresses (e.g., divorce of adoptive parents or the presence of a psychiatric disorder in an adoptive parent), only women with a genetic liability for alcoholism were at increased risk for major depression.

Hormonal status also may be an important modifier of the genetic risk for alcohol involvement, because researchers found that early alcohol use and level of alcohol consumption among female adolescents were associated with the age at first menstruation (i.e., menarche) (Dick et al. 2000). Menarche could represent either a biological or an environmental modifier, because in addition to experiencing hormonal changes, girls who mature early may have more contact with older peers and greater access to alcohol.

### Processes That Mediate Genetic Risk

When talking about genetic risk factors, it is important to realize that genes only carry information that dictates the synthesis and structure of proteins. Proteins do not directly influence or control the behaviors that result in alcoholism. Accordingly, much research currently is focused on identifying the processes that mediate between genes and alcohol-related behaviors.

Family, twin, and adoption studies have been used to study whether familial resemblance for personality characteristics or certain disorders (e.g., anxiety, conduct, and other drug use disorders) may mediate resemblance for alcoholism. As with the studies of depression, the results are generally consistent with the hypothesis that the same genetic risk factors contribute to the risk for alcoholism and the other disorders. However, the findings do not indicate that these disorders directly mediate the risk for alcoholism.

Another approach is to use genetically informative samples to identify characteristics that are more closely related to the biology of alcoholism and may therefore be markers of the genetic risk for alcoholism. Such characteristics, also known as endophenotypes, include certain brain waves (i.e., event-related potentials), a person’s subjective response to alcohol, the degree of impulsivity, and physiological reactivity (see Schuckit 2000). Sex differences in these factors may provide a clue to how the sexes differ in the etiology of alcoholism. For example, patterns of event-related potentials in adolescent boys (but not adolescent girls) who are at high risk for alcoholism because they have alcoholic relatives differed from those of adolescents who are not at high risk (Begleiter and Porjesz 1999).

## Conclusions and Future Directions

Twin and adoption studies have established an important role for genetic influences in the etiology of alcoholism in men. The evidence for genetic influence on alcoholism development in women is less consistent, but interpretation of the literature is complicated by several methodological issues (e.g., small sample sizes). More recent studies suggest a similar degree of genetic influence for men and women. Some research suggests that sex differences exist in the sets of genes that influence alcoholism risk or perhaps in the interactions of genes contributing to alcoholism risk with other genetic or cultural factors. The study of sex differences in factors that mediate and modify genetic risk may provide clues that will aid the development of targeted prevention and intervention efforts.

Recent research has changed from an emphasis on estimating the extent of the heritability of alcoholism to studying the factors that influence or can serve as markers of genetic liability. By identifying specific genes and factors that increase or decrease risk in people with a genetic risk for alcoholism, researchers may gain a better understanding of the mechanisms through which genetic vulnerability leads to the actual development of alcoholism. After specific genes are identified, this information can be incorporated into epidemiologic studies to determine whether these genes account for the sex differences observed in the prevalence of alcoholism and in the patterns of comorbidity.

## Footnotes

1 Unless otherwise noted, the term “alcoholism” here refers to all types of problematic alcohol use, including the clinical diagnoses of alcohol abuse and dependence.

2 In this study, alcoholism was defined as the presence of alcohol abuse and/or alcohol dependence as specified in the American Psychiatric Association’s *Diagnostic and Statistical Manual of Mental Disorders, Fourth Edition* (DSM–IV) (American Psychiatric Association [APA] 1994).
